# External iliac artery injury following total hip arthroplasty via the direct anterior approach—a case report

**DOI:** 10.1080/17453674.2020.1748287

**Published:** 2020-04-02

**Authors:** Ellen Burlage, Jasper G Gerbers, Bob R H Geelkerken, Wiebe C Verra

**Affiliations:** a Department of Orthopedic Surgery, Medisch Spectrum Twente, Enschede;; b Multimodality Medical Imaging M3i Group, Faculty of Science and Technology, Technical Medical Centre, University of Twente, Enschede;; c Department of Vascular Surgery, Medisch Spectrum Twente, Enschede, the Netherlands

An 82-year old man attended our outpatient clinic with symptoms of osteoarthritis of the right hip. Radiographic examination confirmed this diagnosis.

The patient’s medical history included: atrial fibrillation, for which a coumarin derivate was started in 2009, idiopathic thrombocytopenia, with platelet counts of circa 60 × 10^9^ (normal 150–400 × 10^9^), and prostate carcinoma. After transurethral prostate resection in 2016, at the request of the patient, anticoagulant treatment was replaced by ASA (Ascal). Moreover, despite suffering from atrial fibrillation and a low ventricular rate, the patient had chosen not to receive a pacemaker. Ascal was discontinued 7 days before the planned total hip arthroplasty (THA). Therapy to increase platelet function was not administered preoperatively because of low risk of bleeding associated with platelet counts > 30 × 10^9^ (Yang and Zhong 2000).

## Surgical management

**Figure 1.**
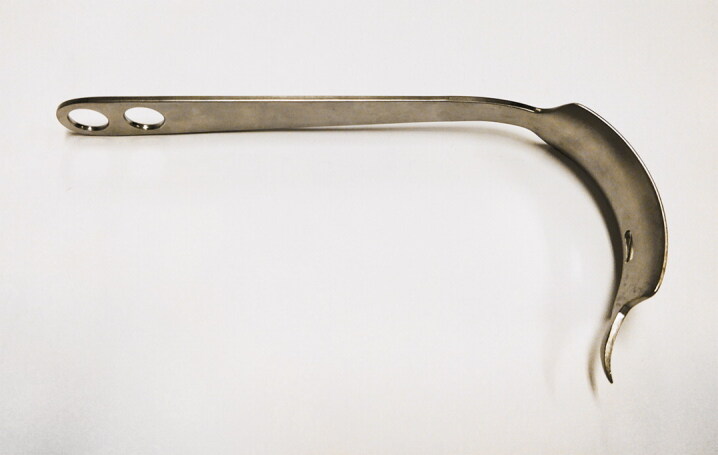
Type of Hohmann retractor used during the procedure.

**Figure 2.**
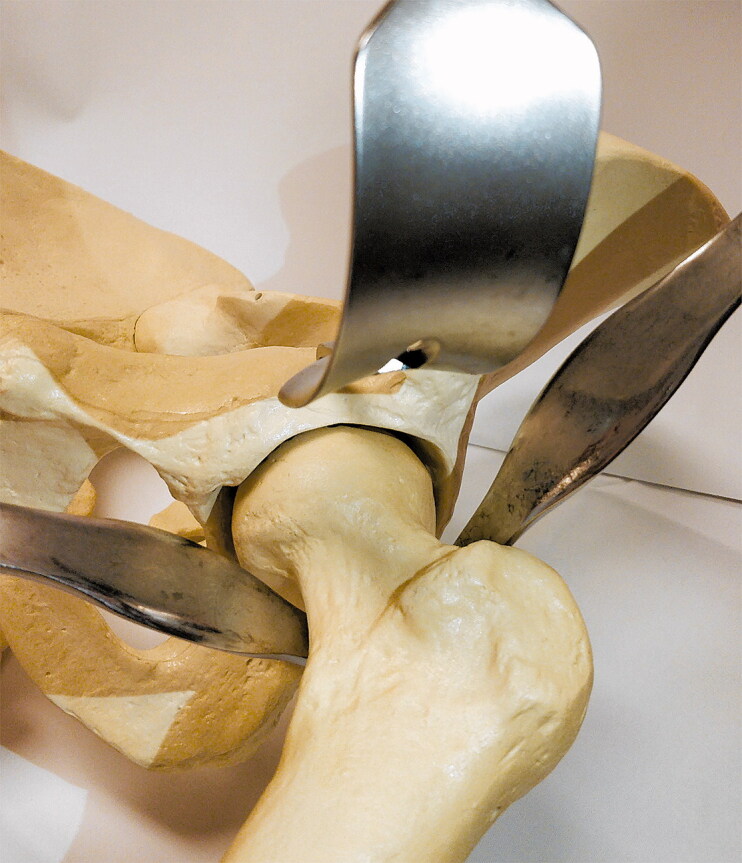
Hohmann retractors placed on a pelvis phantom.

After a period of nonoperative treatment the patient was scheduled for uncemented THA via direct anterior approach (DAA) using spinal anesthesia (Siguier et al. 2004). After incision, the fascia of the tensor fascia lata was incised. The tensor fascia lata and gluteus medius muscles were retracted laterally with a Hohmann retractor (Figure 1), and the sartorius and rectus muscles were retracted medially with a blunt Hohmann retractor in order to expose the anterior hip capsule. The third, pointed, Hohmann retractor was placed under the rectus tendon just at the bony border of the acetabular rim (Figure 2). The retractor aimed in the direction of the contralateral kidney and was fixed using a device that statically holds both the second and third retractor. After opening the anterior capsule, the osteotomy of the femoral neck was performed. There was minimal blood loss, by suction, 50 mL. The labrum was excised and the acetabulum reamed. In the meantime, the patient had become hemodynamically unstable with hypotension, tachycardia and no response to vasopressors and intravenous fluids. In view of the poor circulatory situation, the patient was intubated and cardiopulmonary resuscitation was started.

The surgery was interrupted immediately. The wound was quickly closed with only an implanted acetabular cup and without the use of drainage systems. A transthoracic cardiac duplex ultrasound was performed in the operating theater. There was poor contraction of the anterior wall of the heart. With a high likelihood of acute coronary syndrome, the patient was immediately taken to the cardiac catheterization room. Percutaneous coronary intervention via the right femoral artery showed diffuse coronary artery disease but no significant occlusions. Simultaneously the hematology and biochemistry markers were assessed; the hemoglobin was 3.2 mmol/L (normal 8.5–11.0) and the hematocrit was 0.15 (normal 0.40–0.50). Troponin-T was only mildly elevated at 23 ng/L (normal < 14).

**Figure 3.**
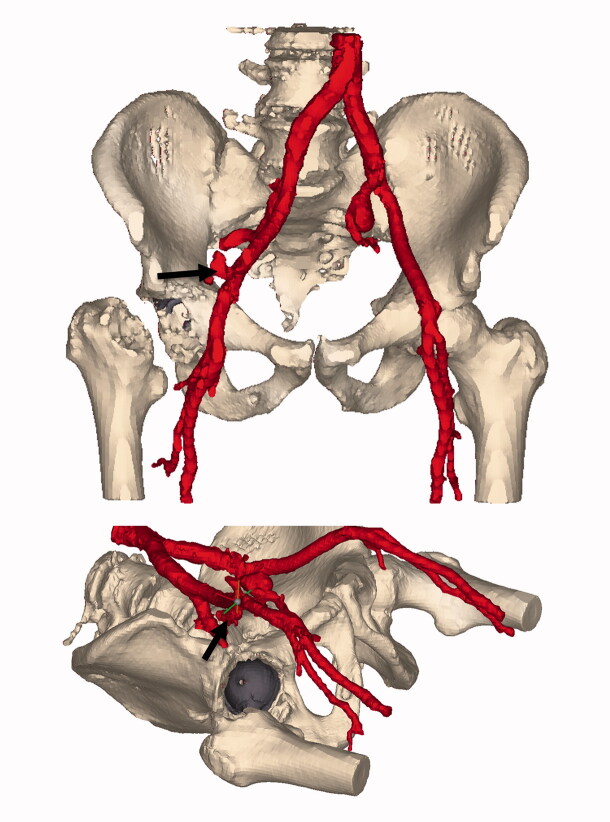
Segmentation of the contrast-enhanced vascular CT. Window ranges corresponding to intravascular contrast are colored red. The implanted metal backed cup is colored gray. The arrow is placed on the site of extravasation at the external iliac artery. Of note is the distance between the acetabular rim and the extravasation site.

Under the suspicion of persisting hemorrhagic shock a multiphase, multislice abdominal aorta-iliac and femoral contrast-enhanced CT scan (ceCTa) was performed. The ceCTa revealed a large retroperitoneal hematoma on the right, with extravasation of contrast in the arterial phase, likely from the distal external iliac artery. Beneath, an asymptomatic left common iliac artery aneurysm with a diameter of 3.5 cm was demonstrated (Figure 3).

The patient was brought back to the operating theater for emergency vascular repair. Due to the uncertainty of the location of the bleeding and the severe hemodynamic instability of the patient an endovascular approach was considered to be less appropriate. The distal aorta and the right common, external, and internal iliac arteries were dissected by a right-sided retroperitoneal approach and a small laparotomy. The distal aorta was controlled, and thereafter the patient was stabilized. Downstream exploration was performed and a nearby circular defect of at least half of the circumference of the external iliac artery was found. The external iliac artery was re-anastomosed end-to-end with Prolene 5-0. The patient was hemodynamically stable and transferred to the intensive care unit. A pacemaker was implanted due to a high-grade atrioventricular block. After 5 days it was considered to be sufficiently safe to finish the THA by implanting the stem of the prothesis. The patient was transferred to the nursing ward within 10 days and discharged to the rehabilitation center on the 16th postoperative day without further complications.

## Discussion

A hemodynamically unstable patient due to external iliac artery injury during a DAA THA has, as far as we know, not yet been described in the literature. 2 recent case reports described common femoral artery lesions, closely related to external iliac artery injury, following THA through DAA (Marongiu et al. 2019, Mortazavi et al. 2019). In recent years THA via the DAA has gained popularity (Siquier et al. 2004, Wang et al. 2018). This popularity of THA procedures being performed via an anterior approach makes awareness of the occurrence of this life-threatening vascular complication important.

Severe vascular injury during total hip arthroplasty is a rare complication estimated at between 0.16% and 0.25% (Nachbur et al. 1979). More recent studies report an incidence of 0.04% in primary THA with an increase to 0.19% in revision arthroplasty (Abularrage et al. 2008). In general, regardless of the surgical approach, injuries have been reported in all the main vessels around the hip, the common femoral artery being the most reported damaged vessel and the external iliac artery thereafter (Shoenfeld et al. 1990, Lazarides et al. 1991). They are at risk because of their anatomical location (Bach et al. 2002, Kawasaki 2012). At the level of the anterior inferior iliac spine the external iliac vessels lie only 7 millimeters from the bone. In some cases they lie directly on the osseous surface as they leave the cavity of the pelvis (Rue et al. 2004, Kawasaki 2012).

Rue et al. (2004) described 2 main groups of vascular injuries during THA surgery: direct and indirect injuries. Direct damage may occur by arterial transection due to a misplaced retractor or by excessive reaming and by arterial penetration of a screw during cup fixation. Longitudinal vascular laceration may cause intraoperative bleeding and a decline in blood pressure. Because the bleeding of puncture injuries results in a slow and small amount of bleeding, it is likely this will not be observed during surgery. The bleeding will form a false aneurysm presenting as a hematoma or pulsatile mass. The patient can complain of hip pain due to pressure or ischemic symptoms caused by impaired blood flow (Proschek et al. 2006). Indirect damage can be caused by compression, stretching, or tearing of a vessel or by excessive heating by the bone cement. Secondary formation of a thrombus or presence of an intimal flap can lead to hypoperfusion and ischemia of the distal leg. Immediately after surgery, the dorsalis pedis arterial pulse can be absent. It is also possible that ischemic pain due to hypoperfusion and the absence of pulsations will not appear until a few hours postoperatively (Mortazavi et al. 2019).

Vascular damage in THA via an anterior approach has been described in 2 recent case reports (Marongiu et al. 2019, Mortazavi et al. 2019). 2 patients had absent distal pulsations immediately after surgery. The 3rd patient had delayed presentation of leg pain and a deficit in the distal arterial pulses. All 3 patients had arterial intimal damage with thrombus formation. Marongiu et al. (2019) reported damage of the common femoral artery caused by misplacement of the anterior retractor. The patient became symptomatic with dropped hemoglobin levels, hematoma, and groin pain a few days postoperatively. In both case reports the femoral artery was involved rather than the external iliac artery as in our case. A systematic review, with 11,810 DAA procedures included, reported 920 complications (fractures, infection, nerve injury, wound complications, dislocation, and revision) (Lee and Marconi 2015). None of these complications had a vascular cause. Alshameeri et al. (2015) performed a systematic review of vascular injuries in association with THA. They identified 124 vascular injuries during the last 22 years, irrespective of the surgical approach. In none of the identified cases was a DAA used.

THA via the lateral and posterolateral approach and their association with vascular injuries has been well described in the literature. Shoenfeld et al. (1990) identified 63 cases via the lateral approach and found the external iliac artery to have the highest injury rate with 36 injuries. Injuries of the external iliac artery consisted of 11 pseudoaneurysms and 17 thromboembolic complications. For the remaining 8 external iliac artery injuries the type of injury was not specified. The causes of the vascular injuries were cement related (one-third), misplacement of a retractor (one-third) or excessive traction on the vessel (one-tenth). Emergent vascular intervention at the time of the THA was necessary in 27 cases. In half of these cases the external iliac artery was involved. The causes of the external iliac artery injury needing emergency intervention were not specified.

The importance in prevention of vascular complications in THA is clear. Alshameeri et al. (2015) reported permanent disability due to ischemia (8%), amputation (2%), and mortality (7%) in 124 vascular injuries as significant consequences of this severe complication. Injury due to retractor misplacement is often described as a cause of vascular damage. It is paramount that patients should be placed in the correct position and that they are handled considering the anatomical location and integrity of the surrounding vascular structures. The neurovascular bundle that contains the external iliac artery travels along the iliopsoas muscle. The iliopsoas muscle bulk will protect the neurovascular bundle against injury (Sullivan et al. 2019). Therefore, the retractor tips should be placed directly on the bone, and the iliopsoas muscle should not be interposed between the retractor and bone (Rue et al. 2004).

Speculation remains as to what caused the active bleeding in our case. The frequently mentioned causes of direct damage to the external iliac artery seem unlikely. Reaming of the acetabulum occurred without complications and thereafter there were no signs of significant blood loss. Neither drills nor screws were necessary to fix the acetabular component. Because the iliac vessels run along the iliacus muscles the retractor has to cross the iliopsoas muscle to cause direct damage to the external iliac artery (Kawasaki 2012, Sullivan et al. 2019). In our case we had no indication during surgery that the Hohmann retractors were located out of the correct anatomical location and we do not believe they were inserted too deep or medially. Considering the type of damage, a nearby circular defect of the artery and repair by end-to-end anastomosis could argue for being a direct cause. The force that esd applied to the external iliac artery could be an indirect cause for the bleeding. Excessive limb manipulation to enact joint dislocation, relocation, and traction by retractors can exert longitudinal stress to the iliac vessels. The external iliac artery is at less risk of tearing due to its thicker intima and flexibility. The presence of atherosclerotic plaques increases the risk of intimal dissection resulting in thrombosis and distal ischemia (Shoenfeld et al. 1990). In our case the hemorrhagic shock occurred as a result of bleeding from the external iliac artery. Therefore the characteristics of this case seem to be less suitable for the pathophysiology of indirect causes of vascular damage.

In conclusion, vascular injury during THA is a rare complication. This complication during DAA has not been described in literature before. Surgeons should be mindful of the fact that injury to the external iliac artery can occur during THA via the DAA, by either direct or indirect means. Last of all it is important to remember that hemorrhagic shock in peracute hemodynamically unstable patients cannot be excluded if there are no signs of significant blood loss in the surgical field.

### Ethics and potential conflicts of interest

Informed consent was given by the patient. The authors declare no conflicts of interest.

## ^Supplementary data^

A video of the contrast-enhanced vascular CT is available at: https://jepper.stackstorage.com/s/GI6wgbB6pP5NBj3
